# *Linum usitatissimum* Delivery over Chitosan Nanobiopolymer: Enhanced Effects on Polycystic Ovary Syndrome Condition

**DOI:** 10.1049/2024/6693566

**Published:** 2024-07-17

**Authors:** Abolfazl Bayrami, Maryam Sojoudi, Shima Rahim Pouran, Aziz Habibi-Yangjeh, Sanaz Sojoudi

**Affiliations:** ^1^ Department of Biology Faculty of Science University of Mohaghegh Ardabili, Ardabil P.O. Box 179, Iran; ^2^ Department of Environmental and Occupational Health Social Determinants of Health Research Centre Ardabil University of Medical Sciences, Ardabil, Iran; ^3^ Department of Chemistry Faculty of Science University of Mohaghegh Ardabili, Ardabil P.O. Box 179, Iran

## Abstract

Herein, chitosan nanoparticle (CHIT) was used as a safe and biocompatible matrix to carry flaxseed (*Linum usitatissimum* L.) extract (FSE). The number of main features and bio-interface properties of CHIT-FSE were determined by SEM, DLS, FTIR, XRD, TGA, and zeta potential analyses and compared to those of chitosan lacking FSE. A GC–MS analysis was also conducted to reveal the bioactive compounds of FSE. The active anchoring of the FSE phytomolecules over chitosan nanoparticles with enhanced thermal and structural stability was correspondingly verified. Subsequently, the influence of CHIT-FSE, CHIT-TPP, and FSE supplementation was assessed on hormonal and biochemical markers of polycystic ovary syndrome (PCOS) in female rats and compared with untreated and healthy control groups. After 16 days of treatment, CHIT-FSE represented the best performance for controlling the serum levels of the studied biochemical (lipid profile and blood glucose level) and hormonal (insulin, testosterone, luteinizing, and follicle-stimulating hormone) parameters. Considering the negligible therapeutic activity of CHIT-TPP, the enhanced activity of CHIT-FSE compared to only FSE was expounded based on the potent action of chitosan nanoparticles in enhanced stabilization, bioavailability, transport, and permeability of the therapeutically important phytomolecules. As per the results of this investigation, supporting medically important biomolecules over chitosan can enhance their therapeutic effectiveness in controlling PCOS.

## 1. Introduction

Chitosan is a natural heteropolymer, which has sparked a great interest in miscellaneous areas of medicine, especially drug delivery studies, on the basis of being biodegradable, biocompatible, soluble, and safe [1]. Hence, a large number of studies are devoted to developing advanced therapeutic drug delivery systems using this macromolecule, which is nontoxic and has a high potential for absorption-enhancing effects [2, 3]. However, attempts are being made to address the inadequate mechanical quality of chitosan under wet conditions, as it restricts chitosan application for carrying therapeutic compounds in the body [4]. In this regard, either some other support materials, such as graphene oxide or cellulose, are added to chitosan, or its mechanical resistance is improved by applying cross-linking molecules such as sodium tripolyphosphate [5, 6]. The electrostatic interaction between the tripolyphosphate anions and the positive surface of chitosan results in a stabilized structure of chitosan hydrogels, which can be successfully engaged in drug delivery applications. Recently, some studies have confirmed that the physical stability of chitosan hydrogels could be improved via loading with phytomolecules of biological origins, e.g., polyphenols. In this instance, not only the opposite charge of the biomolecules improve the stability and surface properties of chitosan, but they also prevent the aggregation of droplets due to the developed repulsive interactions [7, 8]. There are several reports on the combination of chitosan with oils and extracts rich in active biomolecules such as epigallocatechin gallate [9], gallnut extracts [10], henna leaves [11], tea tree oil [12], clove buds essential oil [13], and Plai extract [14]. The phytomolecules play similar roles in stabilizing and reducing metal and metal oxide nanoparticles when present in the reaction medium [15, 16]. Moreover, these bio-compounds enhance the biomedical activities of the green-synthesized nanoparticles [17].

Flax (*Linum usitatissimum* L.) is one of the widely used herbs from the Linaceae family. Being a rich source of pharmaceutically effective ingredients, flax has been extensively cultivated and consumed all over the world for several thousand years [18]. The seed of flax is especially of great interest owing to its constructional proteins (globulins and glutelin), monosaccharide (e.g., L-galactose, D-xylose, L-arabinose), polysaccharides (e.g., D-galacturonic acid, L-rhamnose), polyphenols (e.g., neolignans), phytoestrogens lignans (e.g., matairesinol), essential fatty acids (e.g., alpha-linolenic acid), and various essential minerals (Figure S1). Data from several studies have identified the effectiveness of flaxseed extract in dealing with various health problems such as cardiovascular diseases, hypertension, osteoporosis, arthritis, diabetes, obesity, cancer, and menstrual cycle disturbances [19, 20, 21, 22].

Stein–Leventhal syndrome, which is popularly known as polycystic ovary syndrome (PCOS), is a multidimensional health status that is experienced by a considerable number of females of reproductive age worldwide (4%–20%) [23]. Though the etiology of PCOS is unidentified, the metabolic irregularities (e.g., dyslipidemia and prediabetes conditions) and reproductive abnormality in PCOS patients are clinically interdependent on insulin resistance and hyperandrogenism [24]. Under PCOS conditions, the swift pulse frequency of gonadotropin-releasing hormone (GnRH) brings about elevated levels of LH along with the reduced release of FSH. As a result, the maturation of follicles and the ovulation cycle is disrupted following FSH reduction, while higher LH leads to higher androgen secretion from theca cells of polycystic ovaries [25]. Generally, gonadotropins or metformin are recommended by doctors, which are accompanied by a series of side effects [26, 27]. The past decade has seen the rapid development of herbal medicine as being a natural, effective, and safe alternative to deal with the complications associated with PCOS [28]. Considering the effectiveness of various phytomolecules for managing PCOS complications [29], the safe and smart delivery of these medicinally important bio-compounds is of particular eminence [30].

This investigation aimed to evaluate the efficiency of a chitosan-based delivery system for the safe and effective administration of phytomolecules from Flax (*L. usitatissimum* L.) seed extract in managing complications associated with PCOS. The specific objectives were to (i) characterize the chitosan-based system using scanning electron microscopy (SEM), dynamic light scattering (DLS), Fourier-transform infrared (FTIR) spectroscopy, X-ray diffraction (XRD), thermo-gravimetric analysis (TGA), and zeta potential analyses and apply GC–MS analysis for flax seed extract (FSE); (ii) administer the chitosan-based system to female rats with PCOS and evaluate its impact on hormonal and biochemical factors; (iii) measure the serum levels of total cholesterol (CHOL), total triglycerides (TTG), HDL cholesterol (HDLc), and fasting blood glucose (FBG), along with the hormonal profile, including insulin, testosterone, luteinizing hormone (LH), and follicle-stimulating hormone (FSH), after 16 days of treatment; (iv) compare the effects of treatments with only chitosan (CHIT), only FSE, and the chitosan-extract combination (CHIT-FSE) against control and untreated PCOS rat; and (v) assess the efficiency of the bio-agents in regulating the studied hormonal and biochemical factors.

## 2. Materials and Methods

### 2.1. Chemicals

The chemicals utilized in this research were of analytical grade, excluding repurification. Flaxseeds were provided by a domestic shop (Iran). Chitosan (C_6_H_11_NO_4_)_n_ of low molecular weight (50,000–190,000 Da) with a low degree of acetylation (75%–85% deacetylated) was purchased from Sigma-Aldrich Co. tripolyphosphate ion (TPP, Na₅P₃O₁₀), ethanol (C_2_H_5_OH), and acetic acid (C_2_H_4_O_2_) were purchased from Merck (Germany). A set of specific kits was supplied from Pars Azmoun Co. (Iran) to determine the serum values of CHOL, TTG, HDLc, and FBG. In addition, commercial ELISA kits (Mercodia, Sweden) were used to determine the serum levels of the hormones of concern (testosterone, insulin, LH, and FSH). Ketamine and xylazine (Alfasan, India) were used for the anesthesia of rats.

### 2.2. Experimental Animals

The University of Tehran Animal Center provided female Wistar rats (25, 190–220 g, 8 weeks old). Standard conditions of housing and feeding of rats were maintained during the study. The animal-involved experiments were permitted by the Research Ethics Committees of the University of Mohaghegh Ardabili (IR.UMA.REC.1401.098) and conducted in conformity with the national institutes of health guidelines for the use and care of experimental animals (NIH Publications No. 8023, revised 1978). Five groups, each comprised of five rats, were examined in this study: G-1: named control group, which involved normal healthful rats; G-2: untreated PCOS rats, including PCOS-induced rats received saline; G-3: FSE group, including rats with PCOS received FSE; G-4: CHIT-TPP group, including rats with PCOS treated by pristine chitosan-TPP; and G-5: CHIT-FSE group, including rats with PCOS received chitosan-flaxseed extract. The injected nanostructure suspension to the groups was 1 mL/day for 15 days. It was 50 mg/kg body weight of rats in the G-4 and G-5 and 150 mg/kg for the extract-receiving group, G-3 [31]. An intraperitoneal injection of ketamine–xylazine (3 : 1, 2 mL/1kg) was carried out after the completion of the treatment course to anesthetize the rats. The rats were kept fasted overnight (about 12 hr) before the induction of anesthesia. For the euthanasia of the rats, a special rodent guillotine was used for the decapitation of the anesthetized rats. The main advantage of this method is the quick loss of consciousness in rats. The employed guillotine is kept very sharp and cleaned between decapitations. Moreover, it was performed correctly by a technician who has adequate skill and training in this method [32]. Subsequently, the blood samples were obtained and analyzed.

### 2.3. Vaginal Smear and PCOS Induction

Sampling from vaginal discharge helped to study the sexual cycle of the rats, while the detection of the estrous phase was fulfilled via cellular observation [33]. A single dose of estradiol valerate (2 mg/kg bodyweight in 0.2 mL of sesame oil) was muscularly injected into the rats in the estrous phase to induce PCOS following the method of Anbu and Venkatachalam [31]. The vaginal studies were performed on the 7th, 15th, 30th, and 60th, and the stabilization of the polycystic state was initiated on day 30 and confirmed at the estrus stage through histological observations, as illustrated in Figures S2 and S3.

### 2.4. Flaxseed Extract Preparation

The flaxseeds were washed, dried, and grounded. A 100 g of the flaxseed powder was mixed in 500 mL of ethanol (70%) and held in a water bath of 40°C for 48 hr to prepare the ethanolic extract [31]. The extraction medium was then filtered and condensed as evaporated at ambient temperature. The jelly form extract was preserved at <4°C for the following experiments.

### 2.5. Preparation of Chitosan Nanoparticles

TPP was used as a cross-linking agent to encore FSE phytomolecules over the chitosan and prepare the CHIT-FSE sample [31]. In brief, FSE (1.5% w/v) was magnetically stirred with TPP (0.5% w/v) in a separated vessel and then was mixed with a solution containing chitosan 1% (w/v) and acetic acid 1% (v/v) in a dropwise mode and freeze-dried. The same procedure but without FSE was followed for the synthesis of CHIT-TPP.

### 2.6. Analyses of Collected Serum

The treatment efficiency of the prepared samples was assessed by analyzing the serums of the rats in G-3, G-4, and G-5 on day 16 concerning the levels in G-1 and G-2. The commercial kits provided by Pars Azmun Co. (Iran) were used to determine the hormonal levels, viz. CHOL, TTG, HDLc, and FBG in the serums. The levels of insulin, testosterone, LH, and FSH were found in the serums using ELISA kits (Mercodia, Sweden).

### 2.7. Characterization Tools

The SEM images were recorded to study the shape and size of the samples (LEO1430VP), where slab staining technique was used to prepare the samples [34]. The size distribution of the nanoparticles was established by the DLS-Malvern instrument (Westborough, USA). The functional groups over samples were detected by FTIR analysis (Perkin Elmer Spectrum RX I). For this analysis, the sample and KBr were mixed in a 1 : 10 ratio over the wavenumber range of 400–4,000 cm^−1^. The thermograms of the samples were collected by heating them from ambient to 700°C at the rate of 10°C/min under airflow (TGA-Linseis STAPT1000). The crystal structure of CHIT-FSE and CHIT-TPP were studied via XRD pattern (Phillips X-PERT X-ray). The phytomolecules present in the flaxseed extract were determined using Agilent 7890B/5977A Series GC/MS.

### 2.8. Statistical Analysis

All the presented data were mean values ± standard errors of means (SEOM). One-way ANOVA was applied to calculate the variances among the mean values and analyzed by Tukey's multiple comparison test using IBM SPSS Statistics for Windows, version 20 (IBM Corp., Armonk, N.Y., USA). In the analysis of data, the statistical significance was outlined as *p* < 0.05. The results were statistically represented through error bars as a mean ± SEOM.

## 3. Results and Discussion

### 3.1. Characterization Data of Chitosan

**Figure 1:**
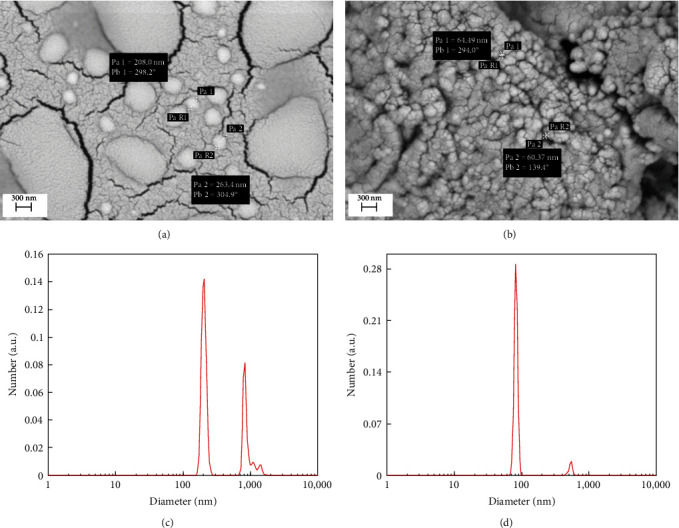
SEM images (a) CHIT-TPP and (b) CHIT-FSE and DLS results of (c) CHIT-TPP and (d) CHIT-FSE.

The morphological study of the TPP cross-linked chitosan and CHIT-FSE was carried out using SEM micrographs (Figures 1(a) and 1(b)). A considerable reduction in the size of chitosan was noted upon FSE charging. It is evident from micrographs that the chitosan hydrogels of about 300 nm were squeezed into much smaller nanoparticles of about 100 nm. Moreover, the prevalent dissimilarity in the size of the CHIT-TPP droplets was controlled mainly due to the stabilizing effects of the loaded FSE phytochemicals [8, 35]. On the other hand, the agglomeration observed for chitosan nanoparticles prepared using flaxseed extract (CHIT-FSE) could be attributed to the presence of extract phytomolecules. These molecules may interact with chitosan or alter the solution properties, affecting nanoparticle formation and surface properties [36].

The size distribution profile of the prepared samples is also studied by DLS. It is evident from Figures 1(c) and 1(d) that the size of the CHIT-TPP droplets varied at two different ranges of 180–320 nm and 6,500–1,200 nm. However, the concurrent supplementation of FSE to the chitosan and TTP in the reaction medium resulted in a considerable reduction in the size of the CHIT-FSE nanoparticles to a range below 100 nm. The polydispersity index (PDI) was also calculated to determine the uniformity degree of the samples based on the size of the droplets. Being a polymer, the PDI of the prepared samples in this study was calculated using the following equation: PDI = *M*_w_/*N*_n_, wherein *M*_w_ and *N*_n_ are, respectively, the molecular weight average and number average molecular weight [37]. In both samples, PDI was 0.5. Based on the DLS results, a PDI of 0.5 was expected for the CHIT-TPP sample, but in the case of the CHIT-FSE sample, this could be attributed to the agglomeration of the nanodroplets.

Zeta potential measurements were also fulfilled, and the values of −23.1 and −26.3 mV were recorded for CHIT-TPP and CHIT-FSE, respectively (Figure S4). The strong repulsion feature between the particles is normally concluded for zeta potential values of −30 or +30 mV [33]. On this basis, a zeta potential of −26.3 mV indicated CHIT-FSE nanoparticles of appropriate stability compared to that of CHIT-TPP. At the same time, the negative values show the negatively charged surface of the droplets, wherein the bigger negative charge of CHIT-FSE indicated a high number of anionic groups of the FSE biomolecules. These anionic groups derived from FSE are supposedly the source of the higher stability of CHIT-FSE in the suspension, which minimizes the agglomeration of the droplets because of the repulsion between the negative charges of surfaces.

**Figure 2:**
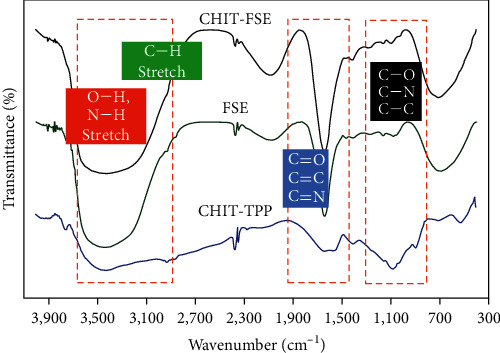
FT-IR recorded spectra derived from FSE, CHIT-TPP, and CHIT-FSE.

A comprehensive FT-IR study was conducted to explore the nature of the interaction between the biomolecules of FSE and CHIT-TPP droplets and also the type of functional groups to reveal the various phytochemicals anchored on the surface of CHIT-FSE. For this purpose, the FTIR spectra of crude FSE, CHIT-TPP, and CHIT-FSE were obtained and presented in Figure 2. The spectrum of crude FSE illustrates a strong broad band ranging from 3,700 to 3,200 cm^‒1^, which belongs to the stretching vibration modes of O‒H and N‒H [38]. The bending modes of N‒H and O‒H also appeared at 1,640 and 1,405 cm^‒1^, respectively [16]. The stretching vibration of C‒H of alkane groups was detected through the band at 2,930 cm^‒1^, while the band corresponding to C‒H bending appeared at 1,412 cm^‒1^. The weak band at 2,100 cm^‒1^ corresponded to the C≡C band, which was adjacent to another strong sharp band at 1640 cm^‒1^ related to C═C/N═C bonds. The C‒O, C‒N, and C‒C bonds were discerned by several small bands ranging from 1,250 to 1,020 cm^‒1^ [39]. The presence of Si–O–C stretching mode could be recognized from the broad strong band at 686 cm^‒1^ [40]. This band could also be assigned to the bending mode of terminal C–H of alkynes (–C≡C–H). In contrast, in the spectrum related to the CHIT-TPP sample, the band expanded from 3,700 to about 3,000 cm^‒1^, corresponding to the O‒H and N‒H stretching vibration modes. The stretching vibration of C–H, C═O, and O–C bonds appeared at 2,929, 1,638, and 1,080 cm^‒1^, respectively. Moreover, the bands at 1,152 and 829 cm^‒1^ originated from the TPP, which are assigned to the stretching vibration of O–P═O and P–O–P modes [41]. Considering the FT-IR signature bands of CHIT-TPP and FSE, the main bands of both samples appeared in the CHIT-FSE sample with dominancy of FSE peaks, indicating the anchoring of FSE biomolecules over chitosan nanoparticles.

**Figure 3:**
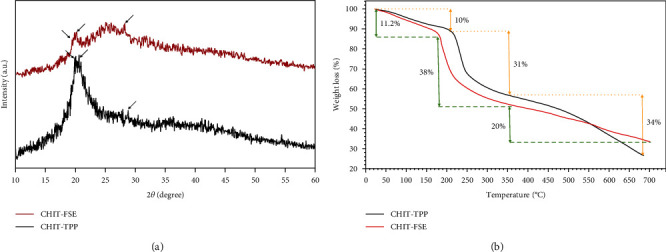
(a) XRD patterns of the CHIT-TPP and CHIT-FSE samples; (b) thermograms of CHIT-TPP and CHIT-FSE samples.

The XRD diffractograms of CHIT-FSE and CHIT-TPP are presented in Figure 3(a). The diffraction peaks at 2*θ* = 19.9° and 20.6° as the fingerprint peaks of crystalline chitosan appeared in the CHIT-TPP sample [42]. Upon biosynthesis process, the corresponding peaks shifted respectively to 2*θ* = 19.5° and 20.2° under the influence of the extract metabolites wherein the coexistence of these biomolecules in the synthesis medium of CHIT-TPP can lead to an amorphous structure [43]. Moreover, the splitting that occurred in these peaks in CHIT-FSE could result from the reduction in the symmetry of the hexagonal phase of chitosan and/or inducing some other phases into its structure. Likewise, the diminution in the intensity of the peaks in CHIT-FSE compared with those of CHIT-TPP could be closely related to the covering effects of FSE biomolecules [43]. Besides, the peak related to the pyrophosphate phase of sodium TPP of CHIT-TPP and CHIT-FSE developed at 2*θ* = 28.8° and 28°, respectively.

Thermogravimetric analysis is a promising indicator of the organic content of CHIT-FSE compared with CHIT-TPP and also the thermal stability of the samples. The weight loss pattern of both samples included a three-step process by which the thermal decomposition of the samples was taken place (Figure 3(b)). In the first step, about 10% and 11.2% of the weight of the CHIT-TPP and CHIT-FSE samples, respectively, vanished up to 150° during the surface H_2_O evaporation course [44]. In the second step, which took place from 180° to 380°, the organic compartments of the samples were thermally broken down, and the decomposition continued as the temperature went up to 700°. As per thermograms, the higher the organic content, the higher the weight-loss percentage. Regardless of the higher weight loss of CHIT-FSE to CHIT-TPP at step 2, the former showed improved thermal stability at higher temperatures primarily derived from the anchored organic moieties.

### 3.2. GC–MS Analysis

The phytochemical composition of the flaxseed extract was revealed using GC-MS analysis (Figure S5). Out of the 118 detected compounds, a list of the compounds with high peak areas is provided in Table 1. Several notable chemical classes, including hydrocarbons, esters, amines, acids, and aromatic compounds, were identified in FSE. For instance, methyl palmitate (hexadecanoic acid methyl ester) and 14-methyl-pentadecanoic acid methyl ester (a derivative of palmitic acid) are fatty acid methyl esters, indicating the lipid content of the flaxseed extract [45]. Moreover, (E)-9-octadecenoic acid and 7-octadecenoic acid methyl esters are unsaturated fatty acids indicative of *α*-linolenic acid as a key intermediate for the synthesis of omega-3 fatty acids [46, 47]. Benzyl nitrile, an aromatic nitrile, can be related to phenolic or aromatic compound metabolism in flaxseed [48]. Detection of 5-decyne and other alkynes shows the diversity of hydrocarbon structures present. The presence of phenolic derivatives was also confirmed by 5-bromovaleric acid, 4-methoxyphenyl ester, and 2,2-dimethylpropanoic acid, 4-methoxyphenyl ester [49]. The compound 2,3,5-trimethyl-1,4-benzenediol (Hydroquinone) was also identified, which is a precursor of vitamin E synthesis [50]. Additionally, some derivatives or fragments of main flaxseed metabolites were detected in the extract (Table 1). This complex mixture emphasizes the diverse bioactive components of flaxseed with nutritional and potential therapeutic properties.

### 3.3. Effects on PCOS Condition

The central aim of this study was to explore the regulatory performance of FSE carried on chitosan nanoparticles on several biochemical (lipid profiles and glucose) and hormonal (LH, testosterone, insulin, and FSH) factors engaged in PCOS. To achieve this goal, the levels of these factors were measured after the induction of PCOS in female rats and also after the treatment course was completed using the studied therapeutic agents, including FSE, CHIT-TPP, and CHIT-FSE samples. The determined values were later compared with the levels in healthy rats and the effects were statistically assessed to signify the regulatory efficiencies.

#### 3.3.1. Regulatory Effects on Lipid Profile

**Figure 4:**
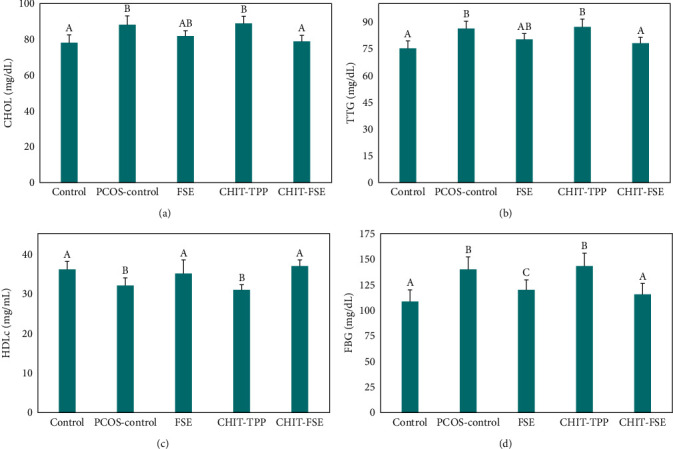
Regulatory effects on (a) total cholesterol (CHOL), (b) total triglyceride (TTG), (c) high-density lipoprotein cholesterol (HDLc), and (d) fasting blood glucose (FBG). (A, B, AB: Insignificant difference between the same letters and Significant difference between the unlike letters (e.g., the differences between control and CHI-FSE were insignificant in all cases, both are marked as “A,” whereas the differences between control and PCOS-control were significant in all cases, one marked as “A” and the other as “B”).

The abnormalities in serum lipid profile, such as the increased levels of CHOL and TTG and decreased levels of HDLc, are generally observed in PCOS patients.  Figure 4 presents the results of the regulatory effects of the studied treatment agents on the serum levels of the lipid profile. From the results obtained for CHOL (Figure 4(a)) and TTG (Figure 4(b)), the differences among the untreated rats with the PCOS group and the groups treated by FSE and CHIT-FSE were significant (*p* < 0.05). Nonetheless, the changes caused by CHIT-TPP were insignificant. Likewise, FSE and CHIT-FSE were effective in enhancing HDLc in PCOS rats to the levels equivalent to the healthy group (Control), to which the HDLc level resulted from CHIT-FSE was 3.8% higher than the control group (Figure 4(c)). On the other hand, CHIT-TPP was not only inefficient in adjusting HDLc level but gave rise to an insignificant reduction compared to that of the PCOS-control group. As per the results of the serum CHOL, HDLc, and TTG of the studied groups, the amount of LDLc could be estimated using the Friedewald formula [51]. The values of 2.7, 3.88, 3.1, 4.06, and 2.64 mg/dL were, respectively, obtained for healthy control, PCOS control, FSE, CHI-TPP, and CHIT-FSE groups, which were in line with lipid profile results. The rich omega-3 (linolenic) and omega-6 (linoleic) polyunsaturated fatty acids (PUFAs) content of flaxseed plays a major role in such cases. Numerous studies continually reported the enhancement in the lipid profile of patients with lipid abnormalities by omega-PUFAs via impeding very low-density lipoprotein (built mainly from cholesterol and triglycerides) synthesis in the liver [52]. Moreover, the flaxseed anthocyanins also participate in lipid profile enhancement by boosting the plasma HDLc level [53]. Considering the superior activity of CHIT-FSE in contrast to unaccompanied FSE, the importance of chitosan in improving the solubility and permeability of CHIT-FSE, together with the targeted bio-availability of biomolecules, should be emphasized. Besides, the anchored secondary metabolites gave rise to further stability of CHIT-FSE, as indicated by the results collected from the zeta potential analysis.

#### 3.3.2. Regulatory Effects on Glucose Serum Level

Another risk factor accompanied by PCOS is hyperglycemia, in which the level of FBG exceeds 126 mg/dL [54]. In the present study, elevated levels of FBG were also detected in PCOS-induced rats. However, CHIT-FSE could successfully fix up the plasma FBG level by 116.5 mg/dL, approaching the control group (109.3 mg/dL). Despite the moderate regulation of FBG level by FSE treatment, CHIT-TPP was ineffective in plasma glucose level regulation (Figure 4(d)). To explain the obtained results, some factors need to be highlighted. In the first place, several secondary metabolites present in FSE fulfill FBG uptake and/or overcome insulin resistance in the patients. For instance, secoisolariciresinol diglycoside, a known phytoestrogenic lignan in FSE, reportedly inhibits the expression of the gene in charge of coding the core enzyme accountable for glucose synthesis in the liver [55]. Moreover, the high percentage of dietary fiber can promote glucose uptake, thus lowering FBG levels in plasma [56]. At the same time, the enhanced bioavailability of the phytomolecules employing chitosan intensified the efficiency of CHIT-FSE when compared with those of only FSE and CHIT-TPP.

#### 3.3.3. Regulatory Effects on Hormonal Imbalance

**Figure 5:**
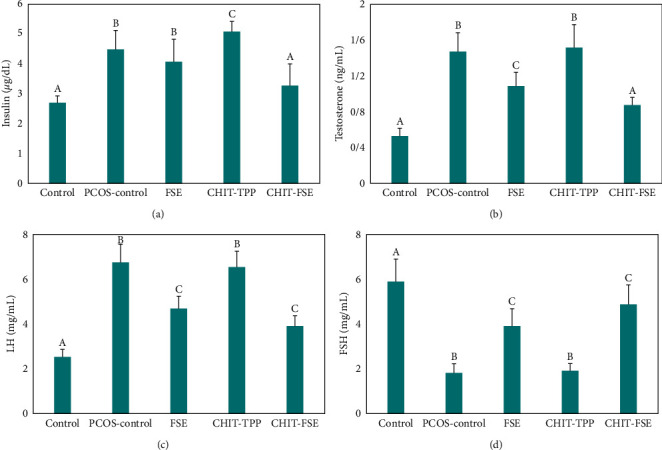
Regulatory effects on (a) insulin, (b) testosterone, (c) luteinizing hormone (LH), and (d) follicle-stimulating hormone (FSH). (A, B, C: Insignificant difference between the same letters and Significant difference between the unlike letters).

Hormonal imbalance is among the main signs of PCOS diagnosis. The levels of the key hormones connected with PCOS condition were measured after the induction of PCOS in rats and also posttreatment.  Figure 5 depicts the changes in testosterone (ng/mL), LH (mg/mL), FSH (mg/mL), and insulin (*μ*g/dL) levels in the studied groups. As stated earlier, PCOS is a multifactor disorder, and its incidence can be confirmed by the concurrent occurrence of several health problems. One of the key features of PCOS is hyperinsulinemia, which emerges following insulin resistance (linked to insulin receptor signaling defects) [57]. Hyperinsulinemia is also a driving force of the ovary's impairment to produce excess testosterone, which in turn aggravates the PCOS condition.

Once plasma insulin exceeds the normal level, the repetition of GnRH is increased, which leads to higher secretion of reproductive hormones (LH and FSH) and an elevated ratio of LH/FSH [58]. Under this condition, the synthesis of estradiol is reduced, and androgens are increased; hence, the follicles are stopped from becoming mature, whereby PCOS is developed. Given this, increasing the sensitivity of cells to insulin can address hyperglycemia and subsequently normalize the metabolism of free fatty acids [59]. In this study, the induction of PCOS increased the amount of plasma insulin in the studied untreated rats by 66.7%. Following the CHIT-FSE therapy for 16 days, this level was mitigated by 27%. However, FSE had moderate (9.3%) and CHIT-TPP showed poor efficiencies (Figure 5(a)). One reason could be associated with lignans, the known phytoestrogens of flaxseed. The familiar lignan of FSE is matairesinol, which has a similar structure to estradiol [60]. Matairesinol has shown significant effects on the plasma level of insulin by decreasing insulin secretion and resistance [61]. Furthermore, flaxseed neolignans are phenolic compounds that protect *β*-cells of the pancreas owing to their antioxidant properties and scavenging of free radicals.

Hyperandrogenism (excess testosterone) was another hormonal disturbance accompanying PCOS-induced rats wherein the plasma testosterone level turned out to be 2.8-fold that of healthy rats. The elevated LH/FSH ratio provokes theca cell proliferation in the polycystic ovary that, in turn, leads to amplified steroidogenesis and eventually contributes to hyperandrogenism. The involvement of various factors, especially genetic factors, has been strongly proven to be connected with anomalous steroidogenesis [62]. The link between insulin resistance and hyperandrogenism is well-studied. The elevated plasma testosterone bioavailability is further provoked following hyperinsulinemia through stimulation of the theca cells of the ovary to produce testosterone and hold the liver back from the secretion of sex hormone-binding globulin (SHBG) [63]. As a result, the concentration of testosterone is amplified in plasma. However, the statuses of the hormones involved in PCOS are interconnected with one another, and the enhancement or reduction of one could give rise to an alteration in the plasma levels of another. For instance, the enhanced levels of LH can provide the basis for increased testosterone production by improving the enzymatic activity of the ovary, which is associated with the fabrication of testosterone. Even though CHIT-TPP had no effects on plasma levels of testosterone, FSE administration reduced the plasma testosterone by 36%, yet significantly higher than the control group. In contrast, CHIT-FSE could successfully diminish plasma testosterone to normal levels with insignificant differences with the control group (Figure 5(b)).

Despite the large number of studies on the development of PCOS, the main pathway underlying PCOS is still unknown. This syndrome is a multifactorial disease, among which is the elevated LH/FSH ratio (i.e., relatively high LH and low FSH concentrations). This hormonal imbalance stems mainly from the aberrant secretion of GnRH associated with GnRH neural network dysfunction [64]. Accordingly, the LH/FSH ratio is generally used for PCOS diagnosis (the ratio is two to three times more in PCOS compared to the normal condition). Therefore, the plasma levels of both hormones were measured in the studied groups and shown in Figures 5(c) and 5(d). As per the results, the plasma level of LH upon PCOS incidence was 2.65 times that of healthy female rats. However, this rate was regulated following the posttreatment by 73% after the course of treatment by CHIT-FSE. On the contrary, the FSE and CHIT-TPP therapies resulted in 44% and 3% reductions in LH levels, respectively. Conversely, under PCOS conditions, FSH levels dropped dramatically wherein the LH/FSH ratio exceeded three-fold than that of healthy rats. Nonetheless, the administration of CHIT-FSE and FSE was also effective in enhancing FSH levels by 2.7-fold and 2.2-fold and normalized the LH/FSH ratio. However, the change in this ratio was insignificant in the case of the CHIT-TPP sample.

**Figure 6:**
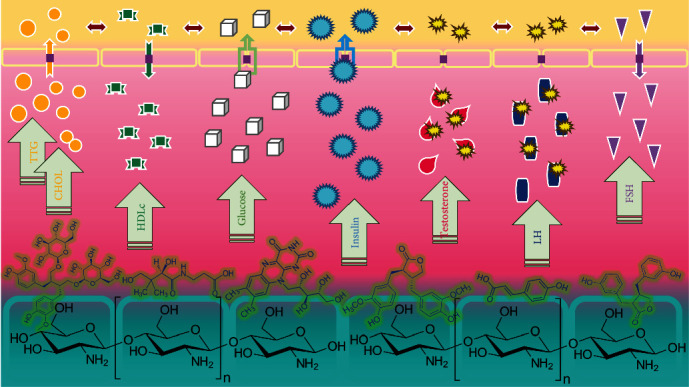
Synergistic action of CHIT-FSE to tackle PCOS and normalize the plasma levels of the studied hormones.

Based on the results described above, FSE performed significantly, while CHIT-TPP had negligible effects on the regulation of the studied hormones. At the same time, the unification of these two therapies gave rise to synergistic effects of which CHIT-FSE could effectively tackle PCOS conditions and normalize the plasma levels of the studied hormones (Figure 6). The synergistic effects of loading extract biomolecules over chitosan on its biomedical activity have been reported over and over [65, 66]. Concerning FSE, the two following factors could have roles in the accomplishment of regulatory goals: (i) Flaxseed phytoestrogens, including enterolactone and enterodiol, are recognized for their regulatory effects on gonadotropins [67]. It is worth noting that flaxseed contains 100 times higher concentrations of lignans when compared to the majority of foodstuffs [68]. (ii) Flaxseed lignans, on the other hand, reduce plasma testosterone further by enhancing the SHBG production by the human hepatoblastoma cell line, HepG2 [69]. As explained above, the phytomolecules' bioavailability, permeability, and safe-guarding are highly valued, which are fulfilled when anchored on chitosan nanoparticles [70]. This will be useful for some other applications as flaxseed extract is a potential antioxidant, antitumor, and anti-inflammatory agent [71].

## 4. Conclusion

In this study, chitosan was loaded with the flaxseed extract phytomolecules and administered to PCOS-induced rats. The results of the characterization analyses evinced that the biomolecules of FSE were actively anchored on the chitosan while enhancing the structural and thermal stability of chitosan. After FSE loading, the size of the chitosan nanoparticles was significantly reduced to nanoranges and its suspension stability was enhanced as per the zeta potential study. On the other hand, the enhanced bioavailability and permeability of phytomolecules of FSE resulted from the potent action of chitosan nanoparticles. As a consequence, CHIT-FSE performed better than FSE in normalizing the hormonal and biochemical variables of the PCOS condition by reducing the CHOL, TTG, FBG, LH, testosterone, and insulin levels and enhancing HDLc and FSH levels to normal values. Altogether, the phytoestrogens could be great elements in regulating the levels of gonadotropins and FBG. Additionally, phenolic compounds of FSE as effective antioxidants could have an important role in the protection of pancreatic *β*-cells against free radicals. Other key phytomolecules of FSE are omega 3- and omega-6- (PUFAs), which could effectively enhance the lipid profile of PCOS patients. As per the results, CHIT-FSE can be considered a potent and risk-free medicine to normalize PCOS conditions in associated women. A number of the limitations that were encountered by this study could be the long time taken for PCOS induction, study group size limitation for ethical considerations, and restricted budget for additional analyses. Similar studies can be designed for further evaluation of the individual secondary metabolites exclusively and in combined form to figure out their roles and activity in controlling the key biochemical and hormonal measures.

## Figures and Tables

**Table 1:** Results of the GC–MS analysis of the studied flaxseed extract.

Area (%)	Ret. time (min)	Peak width 50% (min)	Compound name	Mol weight (amu)
5.72	3.544	0.065	2-Fluoro-2-methyl-propane	76.69

1.68	17.237	0.037	5-Cyano-1,2,3,4-tetrahydro-4,6-dimethyl-2-oxopyridine	150.079
			2-Methyl-5-(1-methylethyl)-phenol	150.104

1.70	17.317	0.042	Benzyl nitrile; m-aminophenylacetylene	117.058

1.20	20.968	0.094	1-Imidazol-1-yl-2,2-dimethylpropan-1-one	152.095
			*N*-(ethoxymethyl)-*N*-ethyl-ethanamine	131.131
			2-Nonenoic acid	156.115

1.46	21.740	0.088	5-Decyne	138.141

2.72	24.104	0.049	*o*-Acetylphenetidine	179.095
			4-Mercaptoimidazo[4,5-c]pyridine	151.02

1.60	24.573	0.048	5-Bromovaleric acid, 4-methoxyphenyl ester	286.02
			2,2-Dimethylpropanoic acid, 4-methoxyphenyl ester	208.11

1.46	25.053	0.053	2H-1-Benzopyran-3-carboxylic acid, 2-ethoxy-2,4-diphenyl-, ethyl ester	400.167

2.72	25.397	0.082	Pipradrol	267.162
			2-Propyl-, (S)-piperidine	127.136
			5-Pentyl-pyrrolidin-2-one	155.131

1.82	25.477	0.05	6,13-Diazadispiro [4.1.5.2]tetradecan-14-one	208.158
			6-Methyl-furo[3,4-c]pyridine-3,4 (1H,5H)-dione	165.043

1.32	25.883	0.054	(p-Butylphenoxy)acetic acid	208.11
			1-Dimethylisopropylsilyloxy-3-methylbenzene	208.128
			*N*, *N*-Dibutyl-4-fluoro-benzamide	251.169

1.45	25.997	0.044	6-Methyl-6-(5-methylfuran-2-yl)heptan-2-one	208.146
			1,2,3,6-Tetrahydro-1-(1-oxobutyl)-pyridine	153.115

1.33	26.266	0.07	1,4-Dimethoxy-2-methyl–benzene	152.084
			2,3,5-Trimethyl-1,4-benzenediol	152.084

1.31	26.633	0.053	2,3,5-Trimethyl-1,4-benzenediol	152.084
			5-Methoxy-2,3-dimethyl-phenol	152.084

5.70	29.551	0.036	Hexadecanoic acid, methyl ester	270.256
			Pentadecanoic acid, 14-methyl-, methyl ester	270.256

7.13	32.320	0.037	(E)-9-Octadecenoic acid, methyl ester	296.272
			7-Octadecenoic acid, methyl ester	296.272

2.44	32.395	0.061	5H-1-Pyrindine	117.058
			*α*-Acetyl-benzeneacetonitrile	159.068

4.37	33.499	0.052	S-[p-Methoxythiobenzoyl]thioglycolic acid	242.007
			2-Aminothiazolo (5,4-b)pyridine	151.02
			1H-Imidazo[4,5-b]pyridine-2-thiol	151.02

2.14	35.439	0.057	2-(4-Methylphenyl)-pyrimidin-4(3H)-one	186.079
			4-Phenoxy-phenol	186.068

1.64	35.794	0.055	3,5-Dihydroxybiphenyl	186.068
			2-Benzyl-2-methyl-5-phenyl-2,3-dihydropyrid-4-one	277.147
			2-Phenoxy-phenol	186.068

2.89	36.091	0.04	8-Hexadecyne	222.235
			Isopropyl linoleate	322.287

2.86	36.165	0.061	(Z)-9-Octadecenamide	281.272

## Data Availability

Data will be available upon request from the authors.

## Abbreviations

ANOVA: Analysis of variance
CHIT: Chitosan
CHIT-FSE: Chitosan-extract
DLS: Dynamic light scattering
FBG: Fasting blood glucose
FSE: Flaxseed extract
FSH: Follicle-stimulating hormone
FT-IR: Fourier-transform infrared
GnRH: Gonadotropin-releasing hormone
G: Group
LH: Luteinizing hormone
GC: Gas chromatography
PCOS: Polycystic ovary syndrome
PDI: Polydispersity index
PUFAs: Polyunsaturated fatty acids
SEM: Scanning electron microscopy
SEOM: Standard errors of means
SHBG: Sex hormone binding globulin
TGA: Thermo-gravimetric analysis
CHOL: Total cholesterol
TTG: Total triglycerides
TPP: Tripolyphosphate ion
XRD: X-ray diffraction
MS: Mass spectrometry.

## Supplementary Materials

Supplementary Materials Supplementary Materials file includes five figures as follows: Figure S1: chemical structure of a number of active phytochemicals present in Flaxseed extract; Figure S2: morphological changes in the phases of the ovarian cycle of the model female rats; Figure S3: polycystic induction confirmation by histological observation of stability at the estrus stage; Figure S4: zeta potential distribution measurements of (a) CHIT-TPP and (b) CHIT-FSE; and Figure S5: compounds detected in the flaxseed extract using GC-MS analysis.
