# Supplementary material for: Linum usitatissimum Delivery over Chitosan Nanobiopolymer: Enhanced Effects on Polycystic Ovary Syndrome Condition
Source: IET Nanobiotechnol. 2024 Jul 17;2024:6693566. doi: 10.1049/2024/6693566 (PMC11268972; doi:10.1049/2024/6693566)
Supplement: Supplementary Materials — file includes five figures as follows: Figure S1: chemical structure of a number of active phytochemicals present in Flaxseed extract; Figure S2: morphological changes in the phases of the ovarian cycle of the model female rats; Figure S3: polycystic induction confirmation by histological observation of stability at the estrus stage; Figure S4: zeta potential distribution measurements of (a) CHIT-TPP and (b) CHIT-FSE; and Figure S5: compounds detected in the flaxseed extract using GC-MS analysis. [file 6693566.f1.docx]

**Supplementary materials**

***Linum usitatissimum* delivery over chitosan nanobiopolymer: Enhanced effects on** **polycystic ovary syndrome condition**

Abolfazl bayrami,^*a^ Maryam Sojoudi,^a^ Shima Rahim Pouran,^*b^ Aziz Habibi-Yangjeh,^c^ Sanaz Sojoudi,^a^

*^a^ Department of Biology, Faculty of Science, University of Mohaghegh Ardabili, P.O. Box 179, Ardabil, Iran.* [abolfazlbayrami@gmail.com](mailto:abolfazlbayrami@gmail.com) [mary.msd701030@gmail.com](mailto:mary.msd701030@gmail.com) [ssd.35a164@gmail.com](mailto:ssd.35a164@gmail.com)

*^b^ Department of Environmental and Occupational Health, Social Determinants of Health Research Centre, Ardabil University of Medical Sciences, Ardabil, Iran.*

[*rahimpooran@yahoo.com*](mailto:rahimpooran@yahoo.com)

*^c^ Department of Chemistry, Faculty of Science, University of Mohaghegh Ardabili, P.O. Box 179, Ardabil, Iran.* [*ahabibi@uma.ac.ir*](mailto:ahabibi@uma.ac.ir)

* Corresponding author:

Tel.: +98 4533514702; Fax: +98 4533514701; Email: [abolfazlbayrami@gmail.com](mailto:abolfazlbayrami@gmail.com)

<https://orcid.org/0000-0001-7002-8605>

* Corresponding author (communicator):

Tel: + 98(45)33534790; Fax: +98(45)33534773; E-mail: [rahimpooran@yahoo.com](mailto:rahimpooran@yahoo.com)

[http://orcid.org/0000-0002-1891-5204](http://orcid.org/0000-0002-1891-5204%20%20%20)

**Fig. S1** Chemical structure of a number of active phytochemicals present in Flaxseed extract (**α-Linolenic acid, Pantothenic acid, Riboflavin, γ-Tocopherol, Nicotinic acid, Secoisolariciresinol diglucoside, Xylose, *p*-Cumaric acid).**

**Fig. S2** Morphological changes in the phases of the ovarian cycle of the model female rats (a) estrus, (b) proestrus, (c) diestrus, and (d) metestrus (Magnification = 40X, scale bar = 100 µm).

**Fig. S3** Polycystic induction confirmation by histological observation of stability at the estrus stage: Histological diagnosis of (a) normal and (b) polycystic ovaries of the studied model rats (Magnification = 40X).

**Fig. S4** Zeta potential distribution measurements of (a) CHIT-TPP and (b) CHIT-FSE.

**Fig. S5** Compounds detected in the flaxseed extract using GC-MS analysis
